# 

**Published:** 1901-01

**Authors:** 


### BOOKS RECEIVED.

Transactions of the State Medical Society of Wisconsin. For the year 1900. Fifty-fourth annual session, held at Milwaukee, June 20, 21 and 22, 1900. Charles S. Sheldon, M. D., Secretary.

Sanity of Mind. A study of its conditions, and of the means to its development and preservation. By David F. Lincoln, M. D., Author of "School and Industrial Hygiene," etc. Duodecimo, pp. 183. New York and London: G. P. Putnam's Sons. 1900. [Price, $1.25.]

Comparative Physiology of the Brain and Comparative Psychology. By Jacques Loeb, M. D., Professor of Physiology in the University of Chicago. Duodecimo, pp. 309. Illustrated. New York: G. P. Putnam's Sons. 1900. [Price, $2.00.]

Appendicitis and its Surgical Treatment. With a report of 185 operated cases. By Herman Mynter, M. D., Professor of Clinical Surgery in University of Buffalo, Buffalo, N. Y. Duodecimo, pp 231. Third revised edition. Philadelphia: J. B. Lippincott Co. 1900. [Price, $2.00.]

The Care of the Consumptive. A consideration of the scientific use of natural therapeutic agencies in the prevention and cure of consumption: together with a chapter on Colorado as a Resort for Invalids. By Charles Fox Gardiner, M. D., Member of the American Climatological Association. Duodecimo, pp. 182. New York and London: G. P. Putnam's Sons. 1900.

Quiz Compends. No. 16. A Compend of Diseases of the Skin. By Jay F. Schamberg, A. B., M. D., Professor of Diseases of the Skin, Philadelphia Polyclinic and College for Graduates in Medicine. Second edition, revised and enlarged. With 105 illustrations. Philadelphia: P. Blakiston's Son & Co. 1900. [Price, 80 cents ]

Obstetric Clinic. By Denslow Lewis, Ph. C., M. D., Professor of Gynecology in the Chicago Polyclinic; President of the Attending staff of Cook County Hospital, Chicago. A series of thirty-nine clinical lectures on practical obstetrics delivered to students and practitioners in Cook County Hospital, Chicago. Octavo, pp. 640. Chicago: E. H. Colegrove, 65 Randolph street. [Price, $3.00.]

Diseases of the Nervous System. A text-book for students and practitioners of medicine. By H. Oppenheim, M. D., Professor at the University of Berlin. Authorised translation by Edward E. Mayer, A. M., M. D., Pittsburg. First American from the second revised and enlarged German edition. Large 8vo, pp. 899. With 293 illustrations. Philadelphia and London: J. B. Lippincott Co. 1900

Therapeutics: Its Principles and Practice. By Horatio C. Wood M. D., LL. D., Professor of Materia Medica and Therapeutics, and Clinical Professor of Diseases of the Nervous System in the University of Pennsylvania; and Horatio C. Wood, Jr., M. D., Demonstrator of Pharmacodynamics in the University of Pennsylvania. Octavo, pp. xxxiii.—850. Eleventh edition, remodeled and in greater part rewritten. Philadelphia and London: J. B. Lippincott Co. 1900.

Food and the Principles of Dietetics. By Robert Hutchison, M. D., Edin., M. R. C. P., Assistant Physician to the London Hospital and to the Hospital for

Sick Children, Great Ormond street. Octavo, pp. 566. With plates and diagrams. New York: William Wood & Co. 1900.

Laboratory Directions for Beginners in Bacteriology. An introduction to practical bacteriology for students and practitioners of comparative and of human medicine. By Veranus A. Moore, B. S., M. D., Professor of Comparative Pathology and Bacteriology, New York State Veterinary College, and of Bacteriology, Cornell University Medical College, Ithaca, N. Y. Duodecimo, pp. 159. Second edition, enlarged and revised. Boston: Ginn & Co. 1900. [Price, $1.05]

Nineteenth Annual Report of the State Board of Health of New York. Transmitted to the legislature February 13, 1899. With accompanying maps. New York and Albany. 1900.

The Treatment of Fractures. By W. L. Estes, A. M., M. D., Director and Physician and Surgeon-in-Chief of St. Luke's Hospital, South Bethlehem, Pa. With numerous original illustrations. New York: International Journal of Surgery Co. 1900. [Price, $2.00.]

Progressive Medicine. Volume IV. 1900. A quarterly digest of advances, discoveries and improvements in the medical and surgical sciences. Edited by Hobart Amory Hare, M. D , Professor of Therapeutics and Materia Medica in the Jefferson Medical College of Philadelphia. Octavo, handsomely bound in cloth, 428 pages, 69 illustrations. Philadelphia and New York: Lea Brothers & Co. [Per annum, in four cloth-bound volumes, $10.00.]

A Manual of Surgical Treatment. By W. Watson Cheyne, M. B., F. R. C. S., F. R. S., Professor of Surgery in King's College, London; Surgeon to King's College Hospital, etc.; and F. F Burghard, M. D. and M. S. (Lond.), F. R. C. S., Teacher of Practical Surgery in King's College, London; Surgeon to King's College Hospital, etc. In seven imperial 8vo volumes, with illustrations. Volume IV., 383 pages with 138 illustrations. Philadelphia and New York: Lea Brothers & Co. 1900. [Cloth, $3.75 net.]

